# Neurophysiological Assessments of Brain and Spinal Cord Associated with Lower Limb Functions in Children with Cerebral Palsy: A Protocol for Systematic Review and Meta-Analysis

**DOI:** 10.3390/brainsci11050628

**Published:** 2021-05-13

**Authors:** Leonard Ubalde, Jing-Nong Liang

**Affiliations:** 1 Interdisciplinary Doctoral Program in Neuroscience, University of Nevada, Las Vegas, NV 89154, USA; ubalde@unlv.nevada.edu; 2 Department of Physical Therapy, University of Nevada, Las Vegas, NV 89154, USA

**Keywords:** cerebral palsy, walking, neuroplasticity, neurophysiology, systematic review

## Abstract

Background: Task-dependent neurophysiological adaptations in people with cerebral palsy have been examined using various techniques such as functional magnetic resonance imaging, peripheral nerve stimulation in order to assess H-reflexes, and transcranial magnetic stimulation. This activity-dependent plasticity is hypothesized to improve specific gross motor function in individuals with cerebral palsy. Although these adaptations have been examined extensively, most studies examined tasks utilizing the upper limbs. The aim of this review is to assess the neurophysiological adaptations of the central nervous system in individuals with cerebral palsy during lower limb functional tasks. Methods: A systematic review and meta-analysis will be conducted to evaluate the neurophysiological changes in the brain and spinal cord associated with lower extremity tasks in individuals with cerebral palsy. We will search within PubMed, MEDLINE, Embase, PsychINFO, and CINAHL using a predetermined search string to identify and evaluate relevant studies. Two independent reviewers will screen these studies against our inclusion criteria and risks of bias, and will extract the data from each study. A third reviewer will be used to resolve any disagreement regarding the inclusion of a study between reviewers. Randomized controlled trials as well as cross-sectional studies published in English 10 years before May 2021 that investigate the neurophysiological adaptations in the brain and spinal cord in people with cerebral palsy will be included if they meet the eligibility criteria. Primary outcomes will include scalar values of fractional anisotropy (FA), H-reflex gains or measures of amplitude, as well as motor cortex (M1) cortical excitability as measured by transcranial magnetic stimulation. Discussion: Since no identifiable data will be involved in this study, no ethical approval is required. Our results will provide insight into the neurophysiological adaptations in children with cerebral palsy, which will be useful in guiding directions for clinical decision making and future development of targeted interventions in pediatrics rehabilitation for children with cerebral palsy. Systematic review registration: The protocol for this systematic review is registered with the International Prospective Register of Systematic Reviews (PROSPERO; registration number: CRD42020215902).

## 1. Background

Cerebral palsy (CP) is the most common disorder affecting posture and motor function in children [1]. The most common cause is from periventricular leukomalacia causing white-matter damage around the third trimester [2,3,4]. Other causes revealed by magnetic resonance imaging (MRI) include brain malformation, infarcts of the middle cerebral artery, or hypoxic-ischemic encephalopathy [3,5]. These injuries may occur during development, during birth, or within the first few years of life. The most common subtype of CP is unilateral spastic CP [5], presenting with hyperreflexia, hypertonia, co-contractions, and difficulty performing independent joint movements in one side of the body [6,7,8]. Up to 41% of children will demonstrate difficulties with crawling, walking, and running [9], consequently impacting development negatively.

Damage to cortical and subcortical centers, descending motor pathways such as the corticospinal tract (CST), and spinal reflex pathways is presumed to be the primary cause of these clinical symptoms [10,11,12]. Functional magnetic resonance imaging (fMRI) is a non-invasive method of indirectly measuring brain activity and can be combined with other neuroimaging techniques such as DTI to examine the alteration in cortical activity and reorganization following a brain injury [13,14,15]. Diffusion tensor imaging (DTI) tractography is a method of reconstructing and examining white matter integrity of the brain structures and corticospinal tracts [16]. At the cortical level, bilateral hemispheric activity and cortical reorganization of the contralesional hemisphere have been observed when performing tasks with the spastic side, indicating that a significant contribution of motor function in children with unilateral CP comes from non-lesioned cortical centers [17]. Additionally, decreased CST integrity in the affected lateral half have been observed in children with unilateral spastic CP [16,18]. 

Similar to findings using fMRI, previous studies using single-pulse transcranial magnetic stimulation (TMS) reported changes in upper-limb cortical motor maps in children with CP [19]. This non-invasive, neurophysiologic technique allows for the observation and investigation of the integrity and signaling strength of these descending pathways [20,21,22]. Stimulation at 90% of maximum stimulator output to the lesioned hemisphere elicited no motor-evoked potentials (MEP) in some children with CP; however, when stimulating the unaffected hemisphere, MEPs in bilateral hands were produced, suggesting that the affected lateral half receives ipsilateral projections from the spared motor cortex [19]. Furthermore, improvements in motor performance after intensive upper extremity tasks have been correlated with increased MEP responses [19].

The spinal cord circuitry is commonly assessed by electrically stimulating the afferent nerve eliciting the H-reflex. Variables commonly reported as functional measures of reflex modulation include peak-to-peak H-reflex amplitudes under comparable background muscle activity, and H-reflex gains defined as the ratio of H-reflex amplitude to average background muscle activity [23,24,25,26]. At rest, while exaggerated stretch reflexes have been reported in children with CP [8], peak-to-peak amplitudes of maximal H-reflexes were comparable to age-matched controls [12]. Furthermore, similar to healthy children, H-reflexes are modulated during gait in children with CP, with increased amplitude during stance and suppression during swing. However, in contrast to healthy children, there is no age-dependent development of H-reflex modulation during gait in children with CP [12]. Short-term treadmill training in children with CP further increased the H-reflex suppression during the swing phase of gait [27]. 

Previously, neurophysiological assessments of the brain and spinal cord in children with CP using DTI fractional anisotropy (FA), fMRI, TMS, or H-reflex testing typically examined changes associated with upper extremities tasks [11,17,28]. To our knowledge, no systematic review or meta-analysis has been performed examining the neurophysiological changes that involved lower extremities. A high-quality review examining the neurophysiological changes following lower extremity interventions targeted to improve functional outcomes may instigate the establishment of future clinical practice guidelines for clinicians and practitioners. Thus, we present our protocol to systematically and analytically evaluate the evidence for alterations to neurophysiology measures associated with lower extremity functional tasks in children with CP as assessed using various non-invasive techniques. 

## 2. Methods and Analysis

### 2.1. Review Question

What are the neurophysiological changes in the brain and spinal cord associated with lower extremity tasks in children with CP? 

### 2.2. Search Strategy

This protocol will follow the preferred reporting items for systematic reviews and meta-analyses (PRISMA) guidelines in order to conduct a systematic review of the literature. The following databases will be searched: PubMed, MEDLINE, Embase, PsychINFO, and CINAHL. The search string and use of Boolean operators will be as followed: “cerebral palsy” AND (TMS OR transcranial magnetic stimulation OR neurophys * OR corticospinal OR h-reflex OR Hoffman * reflex OR fMRI OR functional magnetic resonance imag * OR DTI OR diffusion tensor imag *). Only studies published in English, 10 years from May 2021, will be considered.

### 2.3. Type of Participants

Participants aged five to seventeen will be the focus of this study. This is to include an age-range of children that typically demonstrate adult-like normative values of lower extremity joint angle orientations and alignment [29,30,31,32,33]. Studies with participants who present with spastic hemiplegic, diplegic, or quadriplegic CP will be included in this review. Furthermore, participants will have no existing orthopedic injuries or other pre-existing neurological conditions.

### 2.4. Inclusion Criteria

1. Full-text studies published in English 10 years prior to May 2021.
2. Studies conducted on individuals aged five and older with spastic CP.
3. Studies that investigate the neurophysiological changes in children with CP or age-matched controls with the following assessments: peripheral nerve stimulation to assess the H-reflex pathway, TMS to examine cortical excitability of the corticospinal tract, and fMRI/DTI recordings of fractional anisotropy of the corticospinal tract.

### 2.5. Exclusion Criteria

1. Studies published in a language other than English.
2. Studies that do not include a lower extremity task.
3. Studies that do not screen for individuals for use of botulinum toxin, or anti-spastic medication.
4. Studies that do not include non-neurologically impaired controls for comparison.

### 2.6. Primary Outcomes

Measures of neurophysiologic changes should be reported in eligible studies. These include one of the following measures: FA as measured by DTI, cortical excitability as measured by TMS, and H-reflex gain and/or amplitudes elicited via nerve stimulation. Furthermore, all outcomes should be associated with a lower extremity-specific task. 

### 2.7. Data Management

Articles and their titles and abstracts will be screened by two researchers to determine whether they meet the inclusion and exclusion criteria mentioned above. Any discrepancy of an article will be resolved with a third reviewer until a consensus is reached. Articles will then be imported to a reference management system (EndNote 20), and any duplicates will be removed.

### 2.8. Data Extraction

The main data to be analyzed within the abstract are:

1. Descriptive information regarding the study (e.g., study design).
2. Category of CP examined (e.g., spastic vs. dyskinetic vs. hypotonic).
3. Simultaneous use of an intervention or task involving unilateral or bilateral lower extremities.
4. Neurophysiological assessment to examine the neurophysiological effects of lower extremity interventions.

Full texts of articles will be extracted using the reference management system’s built-in “find full-text” tool. Further quantitative data regarding the aforementioned primary outcomes will be extracted by each reviewer and will be assessed for homogeneity in order to determine if a meta-analysis on each outcome is possible. 

### 2.9. Risk of Bias (Quality) Assessment

1. Articles will be independently screened by two reviewers and full readings of articles will be performed along with cross-examination of the respective Study Quality Assessment Tool from the National Heart, Lung, and Blood Institute [34].
2. Articles will be categorized as good, fair, or poor based on guidelines from the aforementioned assessment tools [34].

### 2.10. Strategy for Data Synthesis

Effect sizes at 95% confidence intervals will be collected to assess the relationships within data, as well as Cohen’s d for estimates of effect size. Quantitative data will be extracted from each article, and a chi-squared analysis will be used to determine homogeneity between observed and expected frequencies. Statistical significance will be set at *p* < 0.05. A narrative synthesis will be written if a meta-analysis is not possible due to the heterogeneity of the studies. 

## 3. Discussion

This systematic review will be the first to present a thorough understanding of cortical and spinal cord function in children with CP. Specifically, we aim to report the neurophysiological adaptations following lower extremity tasks as measured by the H-reflex, TMS, or fMRI fraction anisotropy. As several articles will utilize different functional tasks and neurophysiological tools and measurements, the risk of heterogeneity is high. A narrative synthesis will be formed if heterogeneity proves difficult for the synthesis of a meta-analysis. Final conclusions regarding the implications of neuroplasticity following lower extremity rehabilitation in children with CP will be drawn from this systematic review. Limitations will also be discussed in detail.

Analyses from the review findings will provide insight into the neurophysiological adaptations in the developing nervous system of children with CP, which can be used to guide clinical decision-making and the future development of targeted neurorehabilitation protocols in pediatric rehabilitation to improve motor function in children with CP. Understanding the effectiveness of different lower extremity tasks to enhance neuroplasticity may supplement strategies for therapeutic rehabilitation in this subpopulation, and may be insightful for other neuromuscular and neurodevelopmental disorders. This study meets the criteria for waiver of ethical approval, defined by the Institutional Review Board of University of Nevada, Las Vegas. We will publish the results of our study in a peer-reviewed scientific journal regardless of the outcome.

## Data Availability

Not applicable.
